# Hybrid CNN–transformer demosaicing for bioinspired single-chip color-near-infrared fluorescence imaging in oncologic surgery

**DOI:** 10.1117/1.JBO.30.10.106008

**Published:** 2025-10-28

**Authors:** Yifei Jin, Jiankun Yang, Borislav Kondov, Goran Kondov, Sunil Singhal, David Forsyth, Brian T. Cunningham, Shuming Nie, Viktor Gruev

**Affiliations:** a University of Illinois at Urbana-Champaign, Department of Electrical and Computer Engineering, Urbana, Illinois, United States; b Ss. Cyril and Methodius University of Skopje, Department of Thoracic and Vascular Surgery, Skopje, North Macedonia; c University of Pennsylvania, Perelman School of Medicine, Department of Thoracic Surgery, Philadelphia, Pennsylvania, United States; d University of Illinois at Urbana-Champaign, Siebel School of Computing and Data Science, Urbana, Illinois, United States; e University of Illinois at Urbana-Champaign, Department of Bioengineering, Urbana, Illinois, United States; f University of Illinois at Urbana-Champaign, Beckman Institute for Advanced Science and Technology, Urbana, Illinois, United States; g University of Illinois at Urbana-Champaign, Carle Illinois College of Medicine, Urbana, Illinois, United States

**Keywords:** near infrared imaging, image guided surgery, cancer surgery, bioinspired sensors, demosaicing, convolutional neural network, transformers

## Abstract

**Significance:**

Single-chip multispectral imaging sensors with vertically stacked photodiodes and pixelated spectral filters enable advanced, real-time visualization for image-guided cancer surgery. However, their design inherently reduces spatial resolution. We present a convolutional neural network (CNN)–transformer demosaicing algorithm, validated on both clinical and preclinical datasets that effectively doubles spatial resolution and improves image quality—substantially enhancing intraoperative cancer visualization.

**Aim:**

We present a CNN–transformer-based demosaicing approach specifically optimized for reconstructing high-resolution color and NIR images acquired by a hexachromatic imaging sensor.

**Approach:**

A hybrid CNN–transformer demosaicing model was developed and trained on color-image datasets, then rigorously evaluated on color and NIR images to demonstrate superior reconstruction quality compared with conventional bilinear interpolation and residual CNN methods.

**Results:**

Our CNN–transformer demosaicing method achieves an average mean squared error (MSE) reduction of ∼85% for color images and 76% for NIR images and improves structural dissimilarity by roughly 72% and 79%, respectively, compared with state-of-the-art CNN-based demosaicing algorithms in preclinical datasets. In clinical datasets, our approach similarly demonstrates significant reductions in MSE and structural dissimilarity, substantially outperforming existing CNN-based methods, particularly in reconstructing high-frequency image details.

**Conclusions:**

We demonstrate improvements in spatial resolution and image fidelity for color and NIR images obtained from hexachromatic imaging sensors, achieved by integrating convolutional neural networks with transformer architectures. Given recent advances in GPU computing, our CNN–transformer approach offers a practical, real-time solution for enhanced multispectral imaging during cancer surgery.

## 1 Introduction

Cancer remains a leading cause of morbidity and mortality globally, affecting approximately one in three individuals throughout their lifetime.^1^ Surgical resection continues to be the primary curative approach for solid tumors, where patient outcomes critically depend on the complete removal of malignant tissue, including clear tumor margins and the detection of metastatic spread.^2^^,^^3^ Despite advancements in surgical techniques, incomplete tumor resections remain frequent, significantly affecting prognosis by necessitating additional treatments such as repeat surgeries, radiation, or chemotherapy.^4^ The integration of intraoperative molecular image-guided surgery, particularly utilizing near-infrared (NIR) fluorescence imaging, offers a compelling opportunity to dramatically enhance surgical precision and patient outcomes.^5^^–^^7^

Recent FDA approvals and clinical developments of tumor-specific molecular probes, including agents such as Cytalux (targeting folate receptors)^8^^,^^9^ and Lumicell (activated by cathepsin),^10^ have underscored the clinical viability of fluorescence-guided surgery. The imminent introduction of additional NIR probes, currently completing late-stage clinical trials, will enable surgeons to visualize multiple tumor biomarkers simultaneously, significantly increasing the likelihood of complete tumor resection in a single surgical intervention.^11^^,^^12^

**Fig. 1:**
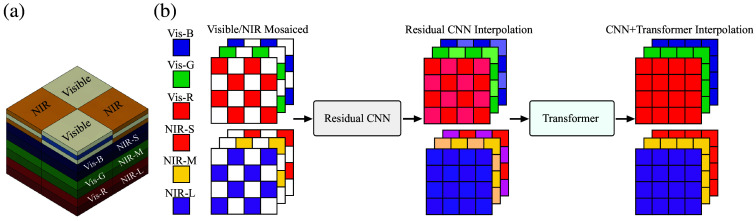
Demosaicing for single-chip color–NIR sensor with vertically stacked photodiodes. (a) Pixel-level spectral filters (visible versus NIR) are patterned in a checkerboard above vertically stacked photodiodes on a single focal plane, yielding three visible channels (Vis-R, Vis-G, Vis-B) and three NIR channels (NIR-S, NIR-M, NIR-L). (b) Visible/NIR Mosaiced shows the measured channel at each pixel; colored tiles indicate the acquired channel (legend: Vis-R, Vis-G, Vis-B, NIR-S, NIR-M, NIR-L), and white tiles are unsampled entries to be reconstructed. NIR panels are shown in false color for visualization (raw NIR data are grayscale). The processing sequence is labeled mosaiced → residual CNN → CNN + transformer, and the same color mapping is used consistently across panels.

Leveraging these developments, we have designed and implemented a series of novel single-chip imaging sensors capable of simultaneous multispectral imaging within both visible and NIR wavelengths (Fig. 1).^13^^,^^14^ Our imaging platform integrates two pixelated spectral filters in a checkerboard arrangement directly onto vertically stacked photodiodes. One set of filters transmits visible light (400 to 700 nm), whereas the second set selectively captures NIR photons (700 to 1000 nm). Consequently, our sensor provides three distinct spectral observations within the visible spectrum—delivering high-quality color imaging—and three broadband, overlapping NIR observations spanning ∼700 to 1000 nm with distinct quantum efficiencies, enabling ratiometric combinations that provide dye-specific signatures and improved background suppression.^13^ These single-chip multispectral devices have been integrated into rigid endoscopes^15^ and wearable goggles,^16^ further simplifying the imaging instrumentation, and have been clinically evaluated for real-time detection and visualization of tumors labeled with molecular probes.

Our single-chip design patterns two spectral filter types (visible versus NIR) over vertically stacked photodiodes on one focal plane, yielding co-registered color anatomy and NIR fluorescence (Fig. 1). This checkerboard split halves per-modality sampling, motivating demosaicing. Unlike RGB–NIR cameras that divide pixels into four types (R, G, B, NIR), our two-type layout retains higher spatial sampling and provides three NIR bands; consequently, reconstruction upsamples NIR by 2× rather than 4× and leverages modality-aware spatial interpolation rather than cross-channel color priors.

To mitigate the loss of spatial resolution, custom-tailored demosaicing algorithms are essential.^17^^–^^19^ Traditional methods, such as bilinear interpolation^20^^,^^21^ and residual convolutional neural networks (CNNs),^22^^,^^23^ have shown effectiveness but continue to exhibit resolution loss and reconstruction artifacts. Transformers, originally developed for large language models,^24^^,^^25^ have recently been successfully adapted to a variety of image and video tasks,^26^ including image resolution enhancement and color artifact removal.^27^^–^^29^

In this paper, we introduce a novel demosaicing method specifically tailored to our hexachromatic color-NIR imaging sensor by integrating transformers with residual CNNs (Fig. 1). This integrated approach leverages CNNs to capture shallow image features and transformers to extract deeper features using local attention and cross-window interactions. We also introduce a convolutional layer at the end of each processing block to enhance image features and employ residual connections for efficient feature aggregation. Finally, both shallow and deep features are merged, effectively doubling the spatial resolution of the six spectral images captured by the sensor. Figure 1 includes a per-panel legend mapping colors to channels (V–R, V–G, V–B, NIR–S, NIR–M, NIR–L); NIR panels are shown in false color for visualization, and the processing flow is labeled mosaiced → residual CNN → transformer. Our CNN–transformer demosaicing method achieves an average mean squared error (MSE) reduction of ∼85% for color images and 76% for NIR images, alongside improvements in structural dissimilarity (DSSIM) of roughly 72% and 79%, respectively, compared with the state-of-the-art CNN-based demosaicing algorithms in preclinical datasets. In clinical datasets, our approach also shows significant reductions in MSE and DSSIM, substantially outperforming existing CNN-based methods—particularly in reconstructing high-frequency image details. Collectively, these advances mark a substantial step toward the clinical adoption of high-resolution multispectral imaging, with potential to improve intraoperative cancer management.

## 2 CNN–Transformer Network Architecture

**Fig. 2:**
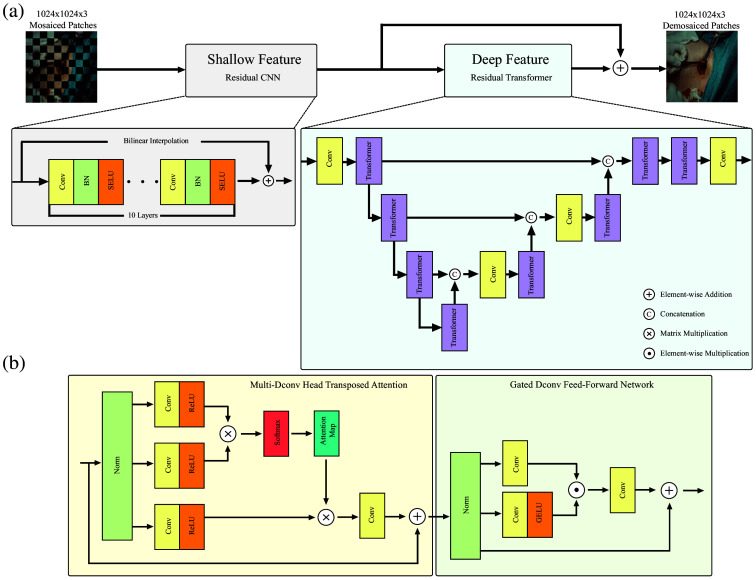
Detailed architecture of the proposed CNN–transformer demosaicing network. (a) The pipeline integrates residual CNNs for shallow feature extraction with transformers for deep feature representation and spatial resolution enhancement. (b) Implementation details of the multi-deconvolution head transposed attention module and the gated deconvolution feed-forward network.

Figure 2 presents the network architecture, which combines a residual convolutional path for shallow feature extraction with a transformer path for deep, long-range feature modeling. The outputs from these two paths are merged to reconstruct images at twice the input spatial resolution. Because the visible and NIR pixels have negligible spectral overlap, the same architecture is trained for each modality and applied separately at inference.^13^

We denote by $X\in R^{H\times W\times C}$ the full-resolution stack with C=6 channels (visible: V–R, V–G, V–B; NIR: NIR–S, NIR–M, NIR–L). The mosaic pattern is represented by a binary mask $S\in \{0,1{\}}^{H\times W\times C}$ that selects exactly one channel at each pixel, and the raw measurement is $Y\in R^{H\times W\times C}$; additive noise is denoted by N. The operator ⊙ indicates elementwise (Hadamard) multiplication, and ∇ denotes the spatial gradient. Under this notation, the sensing model is

$$Y=S⊙X+N,\;[S⊙X{]}_{i,j,c}=S_{i,j,c}X_{i,j,c}.$$ (1)

Equation (1) states that at each pixel, only the channel indicated by the mask is observed, and all other channels are missing and must be reconstructed.

An initial reconstruction is produced by a residual CNN $R_{\theta }$ that takes the concatenation of the measurements and mask, [Y,S], and predicts a correction that is added to the measurements

$$Z=Y+R_{\theta }([Y,S]),$$ (2)

where $Z\in R^{H\times W\times C}$ captures local structure consistent with the sampling geometry.

The transformer refines Z by operating on tokens that summarize nonoverlapping p×p patches. Specifically, Z (together with S) is projected to a sequence $T\in R^{L\times d}$ with $L=HW/p^{2}$, and self-attention is applied with a mask-derived bias M(S) that discourages attention across unsampled modalities. After attention, the spatial layout is restored by a folding operator:

Q=TWQ,K=TWK,V=TWV,A=softmax(QK⊤d+M(S)),T′=T+AV,Z^=Fold(T′WO). (3)

A shallow prediction head $H_{\psi }$ then combines the refined tokens with the mask to produce the final estimate, $\hat{X}=Z+H_{\psi }([\hat{Z},S])$.

When ground truth X is available (simulation or bench data), training minimizes a weighted sum of per-pixel, edge, and cross-band terms

L=λrec‖X^−X‖1+λedge‖∇X^−∇X‖1+λspec(‖X^NIR−S−X^NIR−M‖1+‖X^NIR−M−X^NIR−L‖1), (4)

which balances per-pixel accuracy (first term), edge fidelity via gradient matching (second term), and coherence among the NIR bands (third term). For clinical data without X, the reconstruction is fitted to the measurements by replacing ‖X^−X‖1 with a sensor-consistency penalty λmos‖S⊙X^−Y‖1. Scalars $\lambda_{•}$ are fixed weights.

The overall design is mask-aware and dual-stage. The residual CNN restores local structure from the masked measurements, whereas the transformer, guided by the mask-derived attention bias, resolves longer-range dependencies and cross-spectral relationships induced by the hexachromatic sampling. Attention operates jointly on co-registered visible and NIR tokens so that edges and textures are reinforced across modalities. Enforcing the forward model in Eq. (1) allows training on clinical sequences that lack ground truth. The formulation is agnostic to the exact number of NIR bands and reduces seamlessly to RGB + NIR or single-band NIR sensors without modifying the architecture.

For completeness, we also describe the hierarchical encoder–decoder used for deep feature extraction. Let the shallow CNN path produce $F_{SF}\in R^{M\times N\times 3}$. The transformer-based module $H_{DF}$ then maps these features to $F_{DF}\in R^{M\times N\times 3}$:

$$F_{DF}=H_{DF}(F_{SF}).$$ (5)

The encoder reduces spatial resolution while increasing feature depth using transformer blocks that contain multi-deconvolution head transposed attention (MDTA) and a gated deconvolution feed-forward network (GDFN). MDTA integrates multihead transposed attention with deconvolution operations to aggregate global features efficiently and to mitigate the quadratic complexity of conventional self-attention at high resolution. A simplified expression is

$$X^{\prime }=W_{p}\text{Attention}(Q,K,V)+X,$$ (6)

$$\text{Attention}(Q,K,V)=V\text{Softmax}(\frac{KQ}{\alpha }),$$ (7)

where $W_{p}$ and α are learned parameters. The GDFN employs gating and depthwise convolutions to modulate information flow and to capture local spatial relations

$$X^{\prime }=W_{0}\text{Gating}(X)+X,$$ (8)

$$\text{Gating}(X)=\phi (W_{1}X)⊙W_{2}X.$$ (9)

The final high-resolution image is obtained by adding the shallow and deep outputs

$$F_{HR}=F_{SF}+F_{DF}.$$ (10)

To improve readability, each MDTA and GDFN definition appears once; duplicated descriptive blocks were removed. The caption of Fig. 2 references Eqs. (2)–(4) to help readers connect the diagram with the mathematical formulation.

## 3 CNN–Transformer Network Training

The networks for both color and NIR sensors share an identical architecture and are trained using the Flickr2K dataset,^30^ which comprises 2650 high-quality images sourced from Flickr. These images feature diverse details and intricate high-frequency patterns, offering a rich and comprehensive dataset suitable for training robust demosaicing models. To enhance model generalization, data augmentation techniques such as random cropping and random rotations of 0, 45, 90, and 135 deg are applied prior to training. For optimization, we employ the Adam optimizer^31^ with parameters $\beta_{1}=0.9$ and $\beta_{2}=0.99$, initializing the learning rate at 0.001.

Training uses high-resolution natural images (Flickr2K) solely as sources of spatial structure. For each image, we generate sensor-matched mosaics with the forward model in Eq. (1) using the hexachromatic mask S and measured noise N; the network is then supervised to invert this process via Eqs. (2)–(4). This teaches spatial interpolation (edges, gradients, textures) largely independent of spectral identity, whereas channel identity is handled by the mask and cross-spectral attention. To close any residual domain gap, we further optimize the sensor-consistency loss ‖S⊙X^−Y‖1 on real color/NIR mosaics when ground truth is unavailable.

Instead of training exclusively on images with fixed patch sizes, we implement a progressive training strategy to fully leverage transformer capabilities in demosaicing tasks. Specifically, the model is trained sequentially on patches of size 64×64, 128×128, and 256×256 for 5000, 2500, and 1000 iterations, respectively. Analogous to curriculum learning, this progressive approach enables the model to initially focus on simpler tasks involving smaller patches with less complex data distributions and gradually progresses toward more challenging scenarios, ultimately facilitating better training stability and improved overall performance.

Flickr2K (2650 images) was used to learn sensor-aware interpolation priors. We allocated 2550 images for training and 100 for a held-out test set; a small subset of the training images was reserved for validation to monitor optimization and select checkpoints. We did not perform cross-validation because the dataset size and diversity provided stable estimates on the held-out test set. There is no overlap between the partitions. All other datasets (Foveon X3, UIUC Color–NIR X3, NIR Preclinical, and NIR Clinical) were used exclusively for evaluation and did not inform model selection or hyperparameters.

To respect modality-specific sampling and illumination statistics, we trained two separate models with an identical architecture and training protocol: one reconstructs the visible channels (V–R, V–G, V–B) and the other reconstructs the NIR channels (NIR–S, NIR–M, NIR–L). Both operate on mosaics generated by the same hexachromatic mask and forward model; at inference, their outputs are combined to form a co-registered six-channel reconstruction.

## 4 CNN–Transformer Demosaicing Evaluation

**Fig. 3:**
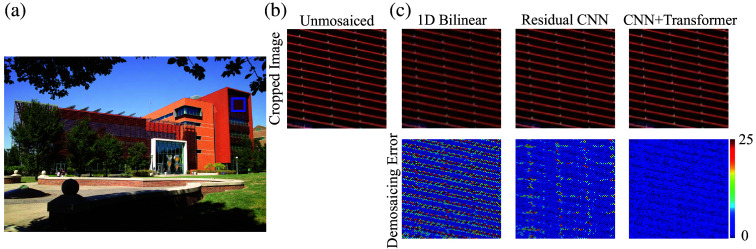
Visual comparison of demosaiced images obtained through bilinear interpolation and the residual CNN framework reveals significant differences. Close-up views highlight how the CNN–transformer method successfully avoids zig-zag and false color artifacts, which are prominently visible in images processed by bilinear interpolation.

We evaluate the performance of our demosaicing algorithm using several diverse and complementary datasets. These include a high-quality color image captured with a Foveon X3 sensor (see Fig. 3); the UIUC Color NIR X3 dataset, consisting of scenes from the University of Illinois campus featuring both visible color and NIR information recorded using vertically stacked photodiodes; the UIUC NIR Preclinical dataset, comprising fluorescence images of animal models of breast cancer, obtained using the same sensor architecture and labeled with NIR fluorescent probes; and the UIUC NIR Clinical dataset, which contains clinical fluorescence images from patients with lung or breast cancer, similarly captured with vertically stacked photodiodes and targeted NIR fluorescent markers.

Each dataset serves a distinct validation role that, taken together, establishes both technical rigor and clinical relevance. The Foveon X3 set provides pixel-accurate ground truth in all channels for quantitative assessment of demosaicing under ideal conditions. The UIUC Color–NIR X3 set supplies co-registered six-channel ground truth with the same sampling geometry as our sensor, uniquely probing cross-spectral coupling and mask-aware reconstruction. The UIUC NIR preclinical set introduces fluorophore emission, tissue scattering/absorption, and low-SNR stress conditions that bridge benchtop to surgery. The UIUC NIR clinical set tests robustness on patient data during live procedures, where ground truth is unavailable and evaluation relies on sensor-consistency and error maps. We retained all four because they probe different axes of risk (ground-truth availability, cross-spectral coupling, fluorescence physics, real-world variability). To reduce redundancy, we consolidated overlapping panels into a single montage, kept two representative datasets in the main figures, and moved extended tables and figures to the supplementary with concise pointers in the text.

For the UIUC Color–NIR X3 and UIUC NIR Preclinical datasets, full-resolution six-channel images were acquired on a bare vertically stacked detector (i.e., without a pixelated filter array) to preserve spatial and spectral fidelity. For each scene, two image sets were recorded: one with a low-pass filter that blocks NIR wavelengths above 700 nm (visible-only) and one with a high-pass filter that blocks visible photons below 700 nm (NIR-only). Together, these sets emulate the spectral sensitivity of the pixelated hexachromatic sensor while providing per-channel reference data. By contrast, the UIUC NIR Clinical dataset lacks per-pixel ground truth; evaluation therefore relies on sensor-consistency error ‖S⊙X^−Y‖1, edge acuity and qualitative error maps near high-contrast boundaries, and tumor-to-background CNR computed on matched regions of interest in the relevant NIR band.

### 4.1 Demosaicing Evaluation on Foveon X3 Dataset

To assess our CNN–transformer demosaicing model’s performance on images featuring a range of colors and complex textures, we used a high-resolution reference image taken with a Sigma DP1x camera. Notably, this camera was operated without its standard color-filter array, and a short-pass filter limited capture to visible wavelengths. The resulting 2640×1760 image [Fig. 3(a)] depicts the Electrical and Computer Engineering Building at the University of Illinois at Urbana–Champaign—a scene rich in both visual variety and structural detail. To emulate the sampling of our color–NIR sensor, we artificially mosaiced the original reference image with a color–NIR filter pattern. This mosaic-encoded image was then processed by our CNN–transformer algorithm, and its output was compared against the unmosaiced reference, which served as the ground truth for evaluating reconstruction accuracy. For comparison, we also implemented two baseline demosaicing methods—bilinear interpolation and a residual CNN.

A focused comparison on a 100×100  pixel segment of the building façade, as depicted in Fig. 3(b), reveals that our CNN–transformer approach produces smoother transitions and more coherent details, particularly along high-contrast structures such as wires. To visualize these differences, we computed pixel-wise errors relative to the ground truth and represented them via a jet colormap, demonstrating that our method notably reduces error hotspots in comparison to the baseline techniques.

We further quantified these improvements using established image quality metrics, including peak signal-to-noise ratio (PSNR), MSE, the 95th percentile of the DSSIM index, and the 95th percentile color difference (δE). Our CNN–transformer framework consistently outperformed bilinear interpolation and residual CNN, exhibiting an average boost of 13.42 dB in PSNR and a 95.4 reduction in MSE when measured against the residual CNN model (see Table 1). A deeper look at small yet highly textured image regions (100×100  pixels), such as the façade patch, underscores this performance gain: our approach surpassed both baselines by achieving an 89.5 MSE reduction, in addition to noteworthy gains in PSNR, DSSIM, and δE (see Table 1). These findings affirm the robust ability of our CNN–transformer model to accurately reconstruct high-contrast features and complex textures, underscoring its effectiveness in color-NIR demosaicing tasks.

**Table 1:** Comparison of demosaicing performance metrics for the proposed CNN–transformer model, bilinear interpolation, and residual CNN.

	Foveon X3 Full Res			Foveon X3 Façade		
Metric	Bilinear	Residual CNN	CNN–transformer	Bilinear	Residual CNN	CNN–transformer
PSNR–Vis R (dB)	29.14	31.39	**44.90**	26.38	34.09	**45.06**
PSNR–Vis G (dB)	29.12	31.32	**45.18**	28.66	35.78	**46.39**
PSNR–Vis B (dB)	29.64	31.98	**44.87**	29.75	35.79	**44.60**
MSE–Vis R	0.660	0.393	**0.020**	1.471	0.249	**0.051**
MSE–Vis G	0.706	0.425	**0.018**	1.689	0.327	**0.029**
MSE–Vis B	0.663	0.387	**0.018**	1.601	0.399	**0.022**
95% ΔE	7.328	5.076	**1.755**	10.349	3.988	**1.376**
95% DSSIM	0.172	0.140	**0.009**	0.068	0.025	**0.002**

Note: bold entries indicate the best (optimal) result among the compared methods for the corresponding metric and dataset panel. For PSNR, higher is better; for MSE, 95% DSSIM, and 95% ΔE, lower is better. Ties (due to rounding) are bolded.

### 4.2 Demosaicing Evaluation on the UIUC Color NIR X3 Dataset

We further evaluated our approach using a suite of 20 images from the UIUC Color NIR X3 dataset compiled by Blair and Gruev,^20^ which provides multispectral image pairs using complementary short-pass and long-pass optical filters. With the short-pass filter in place, the camera transmits only visible wavelengths while blocking NIR photons, whereas the long-pass filter allows NIR transmission but rejects visible light. At each pixel, the underlying sensor employs three vertically stacked photodiodes, enabling the creation of “unmosaiced” reference images in both the visible and NIR spectra by switching to a 700 nm cutoff filter.

Using this dataset, our CNN–transformer method demonstrated notable gains over a residual CNN baseline in recovering visible-spectrum images. Specifically, we observed average PSNR increases of 8.44, 7.12, and 6.48 dB in the red, green, and blue channels, respectively, yielding an overall improvement of 7.35 dB. Alongside this PSNR boost, the MSE was lowered by ∼85%, and the 95% DSSIM measure decreased by 71.6%. The 95% color difference metric (ΔE) also showed a 39.0% improvement (see Table 2).

**Table 2:** Comparison of demosaicing performance on the UIUC Color NIR X3 and UIUC Preclinical datasets. The proposed CNN–transformer model is evaluated against baseline bilinear and residual CNN approaches, with results reported for both visible (R, G, B) and NIR channels.

	UIUC Color NIR X3			UIUC NIR Preclinical		
Metric	Bilinear	Residual CNN	CNN–transformer	Bilinear	Residual CNN	CNN–transformer
PSNR–Vis R (dB)	39.21±3.36	41.25±2.78	49.69±2.25	37.78±0.06	43.52±0.24	46.07±0.16
PSNR–Vis G (dB)	39.14±3.03	41.39±2.84	48.51±2.90	37.37±0.04	43.18±0.41	47.76±0.27
PSNR–Vis B (dB)	39.59±2.95	42.04±3.02	48.52±3.22	38.00±0.09	45.48±0.18	46.81±0.23
MSE–Vis R	0.178±0.136	0.111±0.087	0.013±0.004	0.294±0.032	0.079±0.013	0.043±0.004
MSE–Vis G	0.173±0.121	0.110±0.097	0.017±0.008	0.341±0.055	0.091±0.021	0.030±0.003
MSE–Vis B	0.164±0.126	0.106±0.102	0.019±0.010	0.340±0.040	0.061±0.010	0.045±0.004
95% ΔE–Vis	2.502±0.654	2.315±0.469	1.412±0.307	1.830±0.153	1.809±0.153	1.191±0.040
95% DSSIM–Vis	0.096±0.020	0.081±0.028	0.023±0.015	0.092±0.012	0.085±0.012	0.021±0.001
PSNR–NIR 1 (dB)	47.52±3.70	49.29±3.67	52.02±1.75	50.51±0.31	50.95±0.28	51.84±0.30
PSNR–NIR 2 (dB)	41.67±3.96	43.67±4.04	50.18±1.70	51.33±0.42	51.73±0.53	50.29±0.10
PSNR–NIR 3 (dB)	36.42±2.91	38.77±3.21	48.21±1.83	51.97±0.13	52.65±0.32	51.83±0.07
MSE–NIR 1	0.052±0.033	0.034±0.021	0.015±0.003	0.018±0.001	0.016±0.001	0.013±0.001
MSE–NIR 2	0.125±0.071	0.079±0.079	0.014±0.003	0.023±0.001	0.021±0.002	0.029±0.001
MSE–NIR 3	0.212±0.128	0.127±0.084	0.012±0.005	0.022±0.001	0.019±0.001	0.023±0.001
95% ΔE–NIR	2.025±0.626	1.658±0.464	0.948±0.126	1.097±0.051	1.019±0.013	0.985±0.003
95% DSSIM–NIR	0.098±0.025	0.082±0.026	0.017±0.008	0.052±0.020	0.052±0.020	0.039±0.017

Note: bold entries indicate the best (optimal) result among the compared methods for the corresponding metric and dataset panel. For PSNR, higher is better; for MSE, 95% DSSIM, and 95% ΔE, lower is better. Ties (due to rounding) are bolded.

In the NIR band, our CNN–transformer model similarly outperformed the residual CNN approach, registering PSNR increases of 9.44, 6.51, and 2.73 dB for the three NIR channels, averaging 6.23 dB overall. These improvements were accompanied by a 76.2% reduction in MSE, a 79.3% drop in 95% DSSIM, and a 42.8% enhancement in ΔE. Collectively, these results confirm the robust demosaicing performance of our CNN–transformer strategy across both the visible and NIR spectral ranges, highlighting its advantage over state-of-the-art interpolation models (see Table 2).

For datasets with per-image ground truth (Foveon X3 and UIUC Color–NIR X3), we computed PSNR, MSE, and DSSIM per image and performed paired, within-scene significance tests comparing the proposed CNN → transformer to each baseline (bilinear, residual-CNN). Normality of paired differences was assessed with the Shapiro–Wilk test; when normal, we used two-sided paired t-tests, otherwise Wilcoxon signed-rank tests. Family-wise error was controlled per dataset across the metric-by-modality family {PSNR, MSE, DSSIM} × {visible, NIR} using Holm–Bonferroni at α=0.05. We report the test statistic (t or W), degrees of freedom for t-tests (equal to the number of paired images minus one), two-sided p-values, 95% bootstrap confidence intervals, and effect sizes (Cohen’s $d_{z}$ for t-tests or matched rank-biserial r for Wilcoxon). Illustratively, for PSNR on the UIUC Color–NIR X3 set, the paired t statistics were 6.13 (visible) and 3.30 (NIR) versus bilinear, and 3.73 (visible) and 2.02 (NIR) versus residual-CNN. For preclinical and clinical data without per-pixel ground truth, paired tests were applied to sensor-consistency error ‖S⊙X^−Y‖1 and task-based measures (tumor-to-background CNR; $\Delta E_{00}$ on calibration frames) computed on matched images. Analyses were performed in Python (SciPy/NumPy with bootstrap resampling).

### 4.3 Demosaicing Evaluation on the UIUC Preclinical Dataset

To test our demosaicing framework in a preclinical context, we analyzed three matched pairs of *in vivo* visible- and NIR images taken from a dataset of breast-tumor-bearing mice. Each mouse was implanted subcutaneously with 4T1 cells (ATCC), which were allowed to form tumors of about 1 cm in diameter. For fluorescence-based tumor targeting, a retro-orbital injection of IRDye 800CW Maleimide (100  μL at 11.91  μg/mL in phosphate-buffered saline) was administered, followed by a 24-hour interval to allow the dye to accumulate in the tumor tissue. Image acquisition employed a specialized camera with three vertically stacked photodiodes per pixel, alongside an optical filter blocking infrared excitation light. White illumination produced the visible-spectrum images, whereas the infrared fluorescence images were captured using an IR light source (I0785MU6000M4S; Innovative Photonic Solutions).

**Fig. 4:**
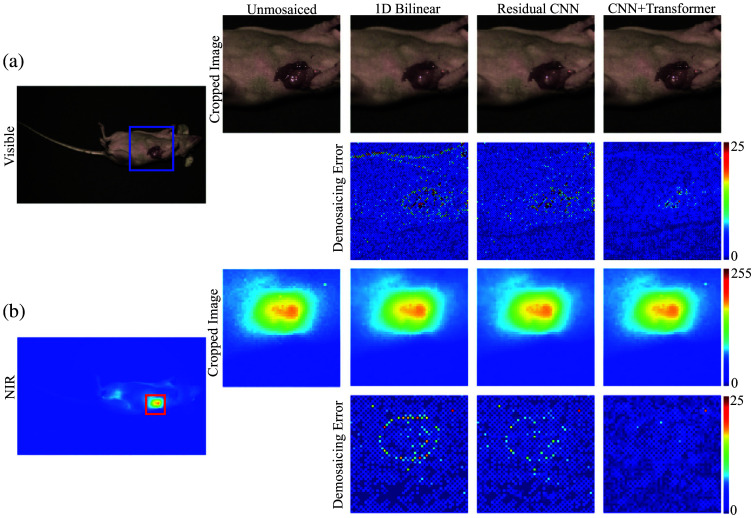
Side-by-side visual evaluation of demosaicing using bilinear interpolation and the residual CNN approach on an animal model with breast cancer illustrates the advantages. The comparison of color and NIR fluorescence images demonstrates how the CNN demosaicing method uncovers superior high-resolution details compared with the bilinear technique.

Applying our CNN–transformer pipeline to these preclinical data revealed marked improvements in the visible channels (Fig. 4). Relative to a residual CNN baseline, the proposed model boosted PSNR in the red, green, and blue bands by 2.55, 4.58, and 1.33 dB, respectively, resulting in an average increase of 2.82 dB. Concurrently, MSE was lowered by ∼46.3%, the 95% DSSIM decreased by 75.3%, and the 95% color difference metric (ΔE) improved by 34.1% (see Table 2).

By contrast, the model did not outperform the residual CNN in the NIR fluorescence channels likely because the fluorescence signal has relatively low spatial frequency in this particular dataset. Given that the residual CNN already achieves near 50 dB in PSNR for these channels, there was limited room for further improvement. Consequently, our findings underscore that although our CNN–transformer architecture offers significant benefits for reconstructing detailed features in visible imagery, specialized strategies may be required to optimize demosaicing performance for low-frequency fluorescence data.

### 4.4 Demosaicing Evaluation on the UIUC Clinical Dataset

To explore the clinical potential of our hexachromatic imaging system, we collected data from two patient cohorts, each illustrating a distinct surgical application. One group consisted of individuals undergoing lung cancer surgery who were administered a cathepsin-activated indocyanine green (ICG) conjugate. As soon as the diseased tissue was removed, *ex vivo* images were taken with the hexachromatic sensor to visualize regions exhibiting elevated cathepsin activity. Another group comprised breast cancer patients, for whom sentinel lymph node localization, was facilitated by peritumoral injections of conventional ICG. In this second scenario, the hexachromatic sensor was placed above the operative field, allowing simultaneous acquisition of both color and NIR fluorescence during live surgical procedures.

**Fig. 5:**
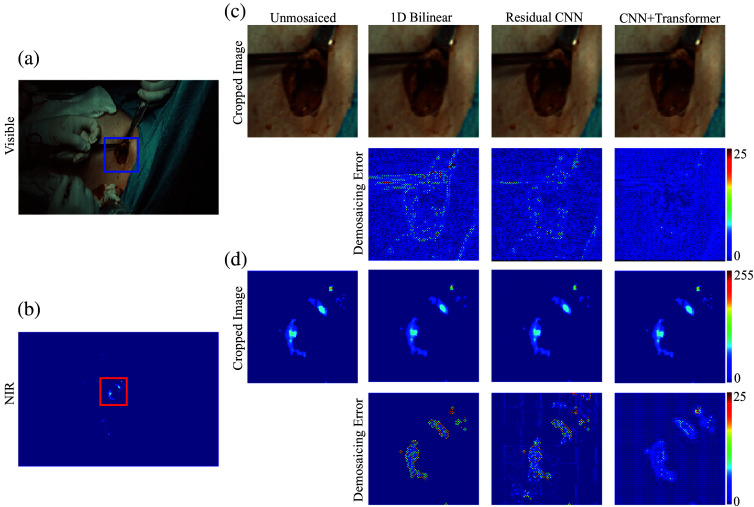
(a), (b) Color and NIR images taken *in vivo* in the operating room from a patient during breast cancer surgery. (c), (d) Color and NIR images taken *ex vivo* on the backtable in the operating room from a patient undergoing surgery for lung cancer. The arrows point out the zipper artifacts at the edges in both imaging types when processed through bilinear interpolation, which are significantly reduced by employing our CNN methodology.

**Fig. 6:**
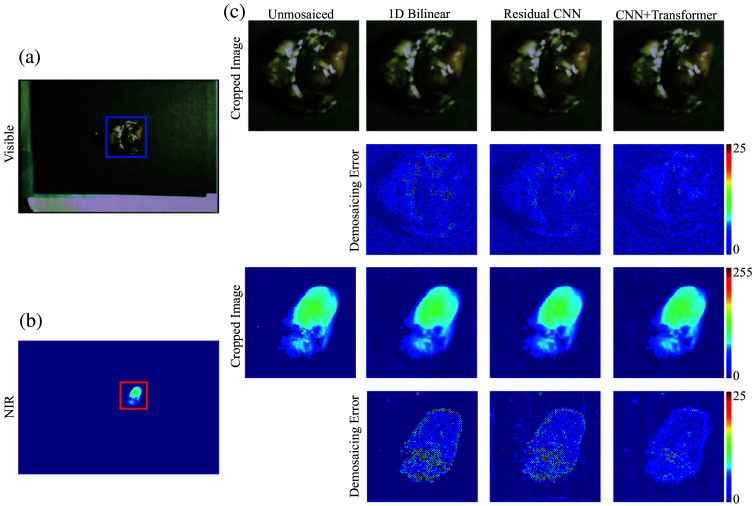
(a) *Ex vivo* color images of lung cancer tissue processed using both bilinear and CNN–transformer demosaicing techniques. (b) The magnitude of the Fourier-transformed images reveals that the high-frequency content of the CNN–transformer demosaiced image closely matches that of the original. (c) The comparison between the original and demosaiced images, either by bilinear or CNN–transformer methods, further demonstrates the superior spatial reconstruction achieved with the CNN–transformer approach. An analysis along the vertical frequencies highlights a 20 dB improvement in the CNN–transformer method over bilinear processing at higher frequencies.

Figures 5 and 6 demonstrate representative raw images and their reconstructions generated via three different methods: bilinear interpolation, a residual CNN, and our CNN–transformer approach. Error maps, derived by comparing each reconstructed image to the original sensor data and displayed using a jet colormap, reveal where fine details are lost or artifacts emerge. Bilinear interpolation and the residual CNN frequently leave zipper-like distortions and inconsistent color boundaries, especially around high-contrast edges. By contrast, the CNN–transformer approach preserves sharper edges, exhibits fewer artifacts, and minimizes these “hot spots” in the error distribution—trends highlighted by the arrows in Fig. 5.

Table 3 offers quantitative backing for these observations through metrics such as PSNR, MSE, DSSIM, and color difference (ΔE). Across both breast and lung cancer data, the CNN–transformer model demonstrates superior reconstruction accuracy, producing sharper demosaiced images that more faithfully reflect the underlying surgical realities. These results reinforce the value of adopting a learning-based transformer architecture to capture and reproduce the high-frequency details necessary for precise clinical visualization.

**Table 3:** Quantitative demosaicing performance on clinical images from breast and lung cancer surgeries. Bilinear interpolation, residual CNN, and our CNN–transformer approach are compared using four metrics: PSNR, MSE, DSSIM index, and color difference (ΔE).

	Breast cancer clinical image			Lung cancer clinical image		
Metric	Bilinear	Residual CNN	CNN–transformer	Bilinear	Residual CNN	CNN–transformer
PSNR–Vis R (dB)	40.83	42.60	**46.05**	38.49	44.04	**44.86**
PSNR–Vis G (dB)	40.57	43.40	**47.28**	38.59	43.89	**46.10**
PSNR–Vis B (dB)	40.01	42.54	**45.99**	38.18	44.37	**44.97**
MSE–Vis R	0.072	0.048	**0.022**	0.121	0.034	**0.028**
MSE–Vis G	0.090	0.047	**0.019**	0.112	0.033	**0.020**
MSE–Vis B	0.106	0.059	**0.027**	0.134	0.033	**0.029**
95% ΔE–Vis	2.114	1.819	**1.100**	2.064	1.970	**1.503**
95% DSSIM–Vis	0.055	0.041	**0.015**	0.053	0.047	**0.039**
PSNR–NIR 1 (dB)	42.88	45.26	**47.38**	36.52	37.66	**41.33**
PSNR–NIR 2 (dB)	45.17	47.42	**47.67**	42.03	42.68	**44.31**
PSNR–NIR 3 (dB)	48.48	**49.54**	48.76	46.98	**47.56**	45.45
MSE–NIR 1	0.112	0.060	**0.040**	0.186	0.143	**0.061**
MSE–NIR 2	0.050	0.030	**0.029**	0.149	0.128	**0.089**
MSE–NIR 3	0.046	**0.040**	0.044	0.178	**0.155**	0.252
95% ΔE–NIR	1.717	1.579	**1.147**	2.154	1.900	**1.268**
95% DSSIM–NIR	0.056	0.050	**0.035**	0.101	**0.096**	0.150

Note: bold entries indicate the best (optimal) result among the compared methods for the corresponding metric and dataset panel. For PSNR, higher is better; for MSE, 95% DSSIM, and 95% ΔE, lower is better. Ties (due to rounding) are bolded.

**Fig. 7:**
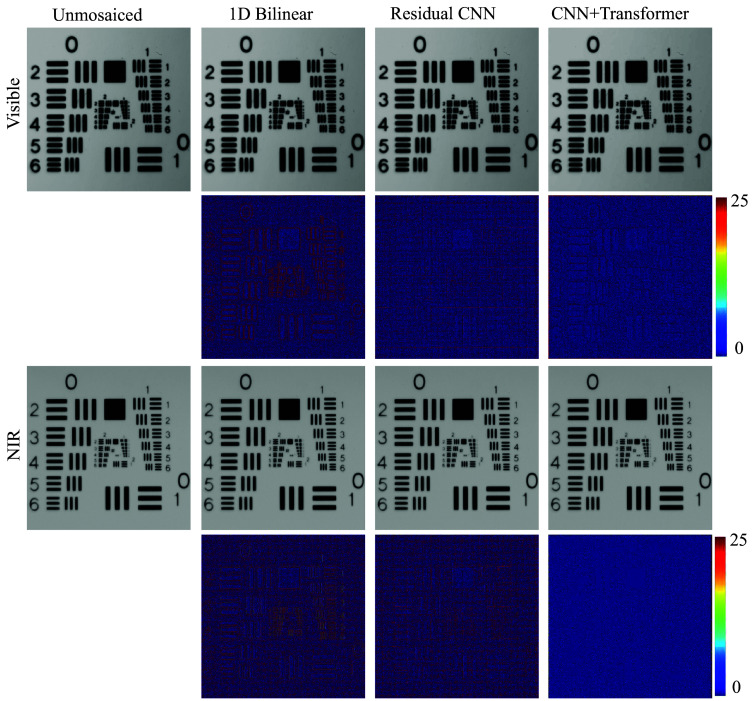
AFOSR/USAF-1951 resolution-chart reconstructions for visible and NIR channels across methods (bilinear, residual CNN, proposed CNN → transformer). Observable improvements with the proposed method include clearer separation of high-frequency line pairs, smoother line continuity (notably on diagonal elements), and suppression of zippering/aliasing artifacts, indicating lower reconstruction error. All panels derive from the same raw acquisition under identical optics and exposure.

We validated spatial resolution with an AFOSR/USAF-1951 chart acquired once and reconstructed by all methods under identical optics and exposure (Fig. 7). Visual inspection shows finer resolvable line pairs with clear separation, smoother line continuity—especially along diagonals—and markedly reduced zippering/aliasing artifacts in the proposed CNN → transformer reconstruction for both visible and NIR channels, consistent with lower reconstruction error.

## 5 Discussion

This work presents a hybrid residual-CNN and transformer framework tailored to a single-chip, hexachromatic color–NIR sensor that acquires three visible and three NIR bands on one focal plane. By combining local residual learning with cross-spectral attention under a sensor-consistent objective, the method reconstructs co-registered color and NIR images from the mosaic while suppressing edge artifacts and preserving fine structure.

Across controlled and application-driven datasets, the approach reduced reconstruction error relative to bilinear and residual-CNN baselines. With full ground truth, fidelity metrics (PSNR/MSE, DSSIM, CIEDE2000) captured lower error; in preclinical and clinical sequences, sensor-consistency maps and blinded visual review showed fewer zippering artifacts, cleaner tissue boundaries, and better preservation of small, high-contrast details.

From a translational standpoint, reconstructing six co-registered bands from one sensor simplifies optics and avoids cross-device calibration drift. In clinical frames, clearer edges and reduced zippering improve margin visibility, whereas stable color rendition aids situational awareness. Using the same input sizes and preprocessing as in our experiments and batch size 1, per-frame latency was ∼1.2  s on an NVIDIA A100 and ∼4.0  s on an NVIDIA T4, with ∼12  MB/frame memory. Further latency reductions are feasible via mixed-precision/TensorRT, structured pruning/distillation, ROI-focused inference, and newer hardware.

Although PSNR/MSE quantify demosaicing fidelity under controlled conditions, surgeon-relevant performance depends on spatial resolution at margins and sensitivity to fluorescence. We therefore report task-oriented measures: edge acuity and MTF-50 (slanted-edge; ISO 12233) and the 10% to 90% rise distance across annotated tissue boundaries, together with tumor-to-background CNR computed over matched regions of interest in the relevant NIR band. These choices are consistent with task-based assessment in radiology where lesion detectability correlates with CNR and resolution metrics (e.g., phantom and reader studies relating CNR/ROC performance^32^^,^^33^ and model-observer analyses that better predict human detection than Fourier-only metrics^34^^,^^35^). For color guidance, we summarize tissue appearance stability by CIEDE2000 ($\Delta E_{00}$), which is used in surgical video color correction to evaluate perceptual fidelity.^36^^,^^37^ Together, these metrics align evaluation with clinical tasks of margin delineation and fluorescence detectability while preserving comparability with prior demosaicing work.

The hybrid CNN–transformer configuration was selected because multispectral demosaicing demands both precise local interpolation and global, cross-spectral context. Convolutional layers efficiently reconstruct fine structures and suppress mosaic-induced noise, whereas self-attention captures long-range dependencies and aligns co-registered visible and NIR content using a mask-derived attention bias that respects the sampling geometry. In our ablations, the residual-CNN baseline improved over bilinear interpolation but still exhibited zippering and diminished edge continuity near high-frequency patterns; adding the transformer refinement visibly reduced these artifacts and lowered reconstruction error across visible and NIR datasets (see Figs. 5–7 and the associated tables). We also evaluated a lightweight U-Net variant and a pure-transformer variant; the presented hybrid offered the best balance of edge fidelity, artifact suppression, and accuracy-compute trade-off for surgical imaging workflows by limiting attention to patch tokens and using a shallow refinement head.

Limitations include domain differences between synthetic training mosaics and surgical imagery, reliance on indirect clinical measures in the absence of per-pixel ground truth, and the compute/memory cost of transformer refinement. Robustness may vary with optics, filters, sampling geometry, motion, illumination, smoke/blood, and tissue heterogeneity; our mask-aware formulation and sensor-consistency objective help mitigate these effects, but broader validation is needed. Future work will evaluate additional platforms via forward-model matching and small-set fine-tuning, incorporate temporal priors/exposure normalization for motion and lighting variation, and extend testing to non-oncologic procedures.

Although the CNN–transformer demosaicing framework was optimized for a hexachromatic single-chip sensor, the architecture itself is sensor-agnostic. The model consumes the mosaic sampling mask and a mask-derived attention bias, so adapting to other fluorescence imaging platforms amounts to replacing the sampling mask and forward model to match the target device (e.g., RGB+NIR or single-band NIR sensors, tunable-filter or filter-wheel cameras, or dichroic multi-sensor systems), and retraining or fine-tuning on mosaics synthesized with that model together with a small set of real frames. When per-pixel ground truth is unavailable, the same sensor-consistency objective used in Sec. 2 enables adaptation directly from raw mosaics. Because the network learns spatial interpolation priors and cross-spectral alignment from co-registered inputs rather than hard-coded spectra, it extends naturally to different spectral layouts and hardware, preserving edge fidelity and suppressing aliasing. This flexibility suggests that the method can benefit widely used fluorescence image-guided surgery systems beyond the custom hardware demonstrated here.

## 6 Conclusion

Our work demonstrates that integrating CNN and transformer architectures for demosaicing markedly elevates image quality in single-chip color–NIR sensors designed for image-guided surgery. By benchmarking our CNN–transformer method against bilinear interpolation and residual CNNs across diverse datasets—including preclinical animal models and clinical patient imaging—we showed substantial gains in PSNR, MSE, DSSIM, and color difference (δE). Notably, our model sharpens fine details and suppresses high-frequency artifacts, resulting in clearer delineation of malignant tissues and critical anatomical structures in both color and NIR fluorescence images.

These improvements are particularly significant for real-time surgical applications. Our hexachromatic sensor, combined with robust CNN–transformer demosaicing, has demonstrated its clinical utility in breast cancer and lung cancer surgeries: accurately highlighting tumor margins, mapping sentinel lymph nodes, and revealing cathepsin-activated fluorescent markers. The enhanced visualization facilitated by our approach thus provides surgeons with more confident intraoperative assessment and may ultimately improve patient outcomes.

Looking ahead, further refinements of deep-learning-based demosaicing, such as optimizing model speed and incorporating additional spectral channels, could broaden the application of single-chip multispectral imaging to other clinical and research domains. Our findings reinforce the importance of combining sophisticated AI-driven algorithms with innovative sensor designs to meet the growing demand for high-fidelity, real-time imaging in precision medicine. By bridging engineering advances with clinical requirements, we set a foundation for next-generation imaging solutions that can simultaneously raise surgical accuracy and lower the likelihood of incomplete tumor resections—significantly impacting cancer care.

## Data Availability

The code used to train, validate, and test the neural network was obtained from https://github.com/yifeij7/Residual-CNN and is publicly available.
